# Research on Multi-Type Rivet Head Defect Extraction and Classification Based on PointGhost Lightweight Network

**DOI:** 10.3390/s26113484

**Published:** 2026-06-01

**Authors:** Liang Liu, Wenxuan Zhou, Xianming Meng, Jianchao Gao, Xinhua Zhao, Ying Zhang

**Affiliations:** 1 Tianjin Key Laboratory for Advanced Mechatronic System Design and Intelligent Control, School of Mechanical Engineering, Tianjin University of Technology, Tianjin 300384, China; 2 National Demonstration Center for Experimental Mechanical and Electrical Engineering Education, Tianjin University of Technology, Tianjin 300384, China; 3 China Automotive Technology & Research Center Co., Ltd., Tianjin 300300, China; 4 Automotive Engineering Corporation, Tianjin 300113, China

**Keywords:** 3D point cloud, rivet head defect, feature extraction, lightweight network

## Abstract

Riveting quality inspection is critical for ensuring structural integrity and safety in aerospace, automotive, and civil engineering, as rivet defects during the riveting process may cause catastrophic failures in structural connections. This study focuses on the detection method for multi-type rivet head defects and aims to improve the performance of feature extraction and classification for various head defects. The research is carried out to develop a lightweight classification network with a Dynamic Screening Self-Attention (DSSA) mechanism for 3D point clouds. To achieve the rivet head dataset, we employ Density-Based Spatial Clustering of Applications with Noise (DBSCAN) clustering to extract each target head data from the dataset of riveted plates. The head dataset can be further simplified using the Non-Maximum Eigenvalue Curvature Method (NMECM). In this way, redundant information can be reduced. The PointGhost network is then designed for the classification of head defects. It contains a sampling module with a Virtual Block Sampling (VBS) mechanism that reduces the computational complexity. In addition, there exists a feature extraction module with a Grouped Pointwise Convolution Ghost (GPC-Ghost) lightweight model that performs local and global feature learning, together with the DSSA mechanism to enhance the riveted head defects. Lastly, the severity levels of rivet protrusion and indentation are quantified using Principal Component Analysis (PCA) and the Total Least Squares (TLS) fitting algorithm. In terms of the experiment, six popular lightweight models are compared, wherein GPC-Ghost shows more significant performance, achieving a 4.31% higher mean accuracy than PointNet++, with less computational cost of 0.66 GFLOPs. Based on the comparative analysis of six attention mechanisms and seven classification networks, the PointGhost model possesses the highest mean accuracy of 99.49%, with an average misclassification rate of 1.19%. The method can balance the accuracy and efficiency effectively, demonstrating its potential for engineering inspection.

## 1. Introduction

As a cold mechanical joining process used to connect lightweight components, Self-Piercing Riveting (SPR) is widely employed in the manufacturing of car bodies and aircraft fuselages [1,2]. The widespread use of SPR technology is expected to reduce energy consumption and emissions. Due to the complexity of the riveting process [3,4], the occurrence of abnormal situations during the forming process will affect the status of the rivets. This may lead to riveting failure, resulting in external or internal defects in the rivets [5,6]. Since the condition of the rivet head can be used to directly assess the reliability of the interlocking structure [7,8], the defect detection for the head plays a critical role in industrial manufacture to ensure the joining quality of products.

Existing methods for riveting defect detection can be classified into three main categories: 2D image-based detection methods, traditional 3D point cloud-based methods, and deep learning-based neural network methods. A comparative analysis is then conducted to evaluate the respective strengths and limitations of the three types of methods.

Firstly, 2D image-based detection is inadequate to present the critical spatial features of rivets [9,10], such as the three-dimensional posture, and quantitative assessments of rivet flush and defects, which are essential for evaluating the riveting quality [11]. Additionally, the detection method based on 2D images is sensitive to illumination and shooting angles, which affect the accuracy and robustness of the detection [12]. While deep learning can be incorporated to raise recognition performance [13,14], the approach remains deficient in depth information and hence hinders the ability to extract sufficient feature information of the riveting status. Consequently, such methods are seldom employed for rivet defect detection.

Secondly, 3D vision technology has been extensively adopted in various defect-detection domains due to its ability to precisely reproduce the spatial information of real targets from point clouds [15,16]. One representative class of methods, which relies exclusively on specific geometric features as opposed to deep learning, processes point cloud data and is therefore referred to as traditional 3D point cloud-based methods. Using 3D vision sensors, high-precision defect detection can be achieved through the extraction and recognition of the geometric contour features of multi-type rivet head defects. Zhou et al. [17] utilized the Density-Based Spatial Clustering of Applications with Noise (DBSCAN) algorithm to extract the point clouds of rivets based on a 3D scanner. The 3D rivet reconstruction was performed by generating localized regions and a gap filling strategy. Based on the contour features of the reconstructed model, the rivet head defects can be identified, such as damage, indentation, and dislodgement. Wang et al. [18] measured the diameter and height of the rivet head with the hybrid data based on 2D and 3D information. The detection accuracy was improved by 50%, and the height measurement error was less than 10 μm, thereby effectively enhancing the defect features. Xie et al. [19] carried out the rivet detection via adaptive density computation with point clouds obtained from a laser 3D scanner. The defects and flush inspection of rivet can be quantified through local density enhancement and the circular fitting method for rivet contours. Although 3D vision detection technology can address the limitations of traditional 2D images, it is advisable to further explore a specific lightweight and efficient defect recognition and classification network to facilitate industrial applications. Therefore, reasonable data-processing methods and defect feature-extraction techniques are key to the identification of riveting defects.

Thirdly, deep learning-based neural network methods have emerged as the dominant approaches in defect detection. Such methods can be combined with existing 3D point cloud processing techniques. These techniques can be categorized into voxel-based methods [20], multi-view based methods [21], and point-based methods [22]. The voxel-based and multi-view based methods may miss feature details during processing, resulting in decreased detection accuracy. By contrast, the method based on original point clouds can preserve complete geometric feature information to achieve high-precision defect detection and hence is widely used. As an innovative achievement, PointNet directly processes unordered 3D point sets using a network architecture based on three 1D convolutions and max pooling, extracting global features for classification [23]. However, when point sets contain complex geometric structures, rich local features must be captured to boost the model’s generalization performance. To this end, PointNet++ is developed by adopting a hierarchical approach—passing through sampling, grouping, and feature-extraction layers in sequence—to learn local features [24]. In this way, it can achieve high-precision classification and segmentation. Subsequently, Ma et al. [25] constructed the PointMLP model based on a lightweight geometric affine module and multiple residual MLP modules. By mapping local point features to a unified scale and extracting local features in a staged manner, the classification performance can be further improved although at the cost of an increased parameter count and computational cost. Furthermore, a non-parametric network termed Point-NN is established based on a non-parametric encoder and a point-memory bank [26]. It can achieve favorable performance on different 3D tasks without training. Based on their foundational framework, Point-PN is further developed with learnable linear layers, which reduces the number of parameters and improves feature learning efficiency.

Although the point-based deep learning methods can extract local geometric features, challenges remain in fully extracting key features. For this reason, attention mechanisms have been incorporated into deep learning architectures to enhance the extraction of subtle features in image processing [27]. It allows models to selectively focus on key input features and capture relationships across different dimensions effectively by offering global perception and flexible interaction capabilities [28,29,30]. Zhang et al. [31] presented an MA-SPRNet model for defect detection of riveted joints. The model is integrated with a multi-attention mechanism that enhances the defect information captured from channel, spatial, and structural dimensions. While the riveting detection precision is improved, the computation load is high. Huang et al. [32] proposed a global attention mechanism based on YOLOv5 to capture defect features on the aircraft body surface and reduce redundant scene information, significantly improving defect-detection performance. Xie et al. [33] introduced a field attention unit to learn the characteristics of the rivet region of the fuselage surface by assigned weights. Based on 3D point clouds, the rivet point prediction is carried on with the RRCNet model, and the accuracy can reach 93.0%. Tang et al. [34] proposed the SCA-Net, a spatial and channel attention-based model that captures the geometric relationship between point cloud patches. The network extracts local features from three divided patches of the dataset and learns their geometric correlations. Then, it extracts global features via auto pooling. Finally, classification and segmentation experiments validated its performance.

It is well known that the learning network with attention modules can improve the classification accuracy. However, this may increase the number of parameters and computational complexity. Therefore, many researchers focus on the design of a lightweight network to improve detection efficiency [35,36]. The prior developed lightweight network MobileNet reduces the parameter computation volume by introducing depthwise separable convolution, which trades off between computation and accuracy [37]. In contrast, the GhostNet model replaces conventional convolutional layers with cheap linear transformations and identity mappings based on the Ghost module [38]. This flexible embedding method with stacked modules is beneficial to promote detection efficiency. Mohammadi et al. [39] designed a Point-LN classification network with non-parametric position encoding, combining the non-parametric components with a streamlined learnable classifier. Based on the ModelNet40 dataset, the conducted classification tests show that Point-LN can offer better accuracy and efficiency simultaneously, for the reduced number of parameters and less runtime. Peng et al. [40] replaced the depthwise separable convolution in the Ghost convolution with DO convolution, thereby constructing the DC-Ghost module, which enhances image features and reduces network parameters and computational costs. Zhang et al. [41] proposed a lightweight position-recognition network, termed LR-Net. It obtains rotation-invariant features with low-dimensional features extraction structure, thereby improving recognition efficiency and accuracy. Recent studies have also explored lightweight 3D point cloud frameworks for edge deployment and industrial inspection applications. To reduce model parameters and computational complexity for deployment on edge devices, Li et al. [42] proposed a lightweight 3D point cloud object-detection architecture tailored for computationally constrained platforms. Liang et al. [43] proposed a Rotationally Invariant Features (RIF) framework for 3D anomaly detection and designed a lightweight Convolutional Transform Feature Network (CTF-Net) to achieve efficient and robust point cloud feature extraction, demonstrating its potential for industrial inspection applications. Despite these advances, a general approach to feature extraction and recognition has not been formulated for various practical applications. There is little research on comprehensive detection methods for various rivet head defects based on 3D point clouds.

Systematic research on defect detection for a multi-type rivet head remains limited in the relevant literature, and even fewer efforts have been devoted to lightweight network architectures that enable efficient defect detection in industrial settings. In fact, the multiclass classification offers an ideal framework for rivet defect recognition, provided that the defects can be learned effectively [44]. To this end, this study proposes a lightweight classification network, PointGhost, for automatic rivet head defect detection based on 3D point clouds. This research contains the following three contributions:

- We create a dataset containing five types of rivet head defects collected by a 3D scanner. The DBSCAN clustering method is first employed to extract individual rivet head data from the riveted plates. Then, the dataset of the rivet head can be formed using the Non-Maximum Eigenvalue Curvature Method (NMECM), which retains the defect features with reduced redundant information.
- As a lightweight network, PointGhost is formulated for defect classification of the rivet head. The developed framework includes three main modules. In the sampling module, a Virtual Block Sampling (VBS) mechanism is proposed to reduce computational complexity. In the feature extraction module, a lightweight model, Grouped Pointwise Convolution Ghost (GPC-Ghost), is introduced for local and global feature learning. In addition, an efficient Dynamic Screening Self-Attention (DSSA) module is proposed to integrate and improve the feature expressiveness for defects. Through these means, multi-type head defects can be classified.
- The severity levels of rivet protrusion and indentation defects are further quantified using the Principal Component Analysis (PCA) method and Total Least Squares (TLS) plane fitting algorithm.

The remainder of this study is organized as follows: Section 2 introduces the type of rivet head defects and presents the riveting samples for detection. This section also proposes an efficient rivet data-extraction algorithm based on DBSCAN clustering. Section 3 introduces our detection architecture. Section 4 describes the experimental research, including the dataset, performance analysis, and flush evaluation of the rivet head. Section 5 summarizes the conclusion and introduces the direction of future work.

## 2. Data Preprocessing

The common defect types of rivet head are first proposed in this section. The specimens are then prepared with steel semi-hollow rivets including the custom defects. In addition, the data-extraction and reduction algorithms based on point clouds are introduced to improve the detection efficiency for subsequent network classification for multi-type rivet head defects.

### 2.1. Defect Types and Specimens

**Figure 1:**
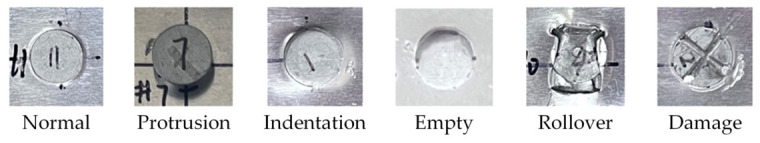
Types of rivet head status.

**Figure 2:**
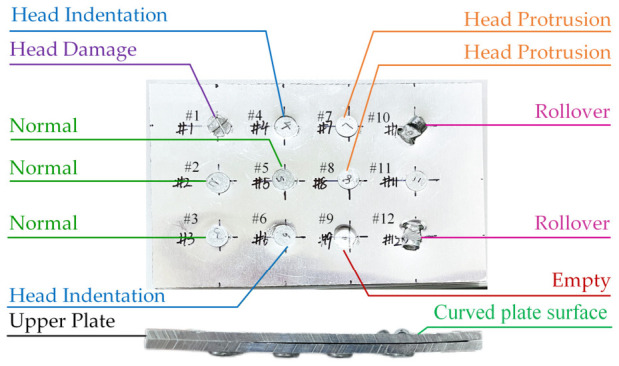
Riveted specimen.

During the riveting process, there are five common types of external riveting defects: head damage, head protrusion, head indentation, empty rivet, and rivet rollover, as shown in Figure 1. Since these defects are closely related to the condition of the rivet head, defect detection can be performed by extracting the point cloud Data of Individual Rivet Head (DIRH) and its surrounding areas on the upper riveted plate based on 3D vision equipment. To this end, specimens were prepared with steel semi-hollow rivets (rivet head diameter: 7.8 mm) and a two-layer 3003 aluminum alloy structure with a total thickness of 6 mm. The specimens contained normal rivet heads and the five types of rivet head defects. Each specimen measured 110 mm in length and 70 mm in width. Figure 2 shows the front and side views of a typical sample.

As for head protrusion and indentation, a flush measurement enables the further classification of defect severity into mild, moderate, and severe levels. The head damage refers to the presence of significant scratches or a partially missing rivet head. According to common rivet inspection standards [45,46], the defect size ranges and severity levels are summarized in Table 1. It should be noted that each of the prepared specimens and the rivets from different specimens are unique. This uniqueness arises from three factors: (1) independent mechanical formation processes, where each rivet undergoes a distinct mechanical force–displacement curve during the riveting process, including variations in the stress, clamping force, and potential operator errors. Consequently, the final 3D morphology of each rivet across different specimens is determined by its independent physical formation process, rather than by a shared specimen-level characteristic; (2) random and diverse defect distributions, meaning that adjacent rivets often exhibit completely different defect types with no systematic correlation; and (3) geometric independence due to the curved plate surface, whereby the riveting process with different partial support arrangement causes the two plates to undergo progressive deformation. After each rivet is riveted, the geometric morphology of the plates changes incrementally, and the varying partial support conditions give each rivet a unique local spatial pose (e.g., distinct normal vectors, point cloud orientations, and curvature distributions). The purpose of this riveting process design is to avoid any potential data leakage to the greatest possible extent.

### 2.2. Data Extraction

**Figure 3:**
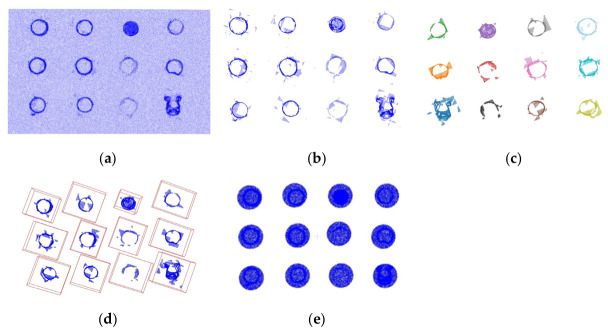
Extraction of single rivet head data. (**a**) Plate data; (**b**) plane elimination; (**c**) rivet clustering analysis; (**d**) frame selection based on axis-aligned bounding boxes; (**e**) rivet head extraction.

To identify the defects of the rivet head, it is essential to extract the contour data of a single rivet on the upper plate. In this paper, the plate surface is scanned by a 3D scanner with a scanning accuracy of 0.05 mm. In this way, the number of sampling 3D points usually exceeds 500,000. Therefore, an efficient algorithm is proposed for extracting rivet head data based on DBSCAN clustering. Its process is shown in Figure 3.

Firstly, all acquired 3D points shown in Figure 3a are divided into *n* × *n* subregions along the *x* and *y* directions, respectively. Secondly, the Random Sample Consensus (RANSAC) algorithm is applied to fit planes for each subregion based on the related points within the region. The point-to-plane distances for the associated points are calculated. Only the specific points with the distance exceeding 0.03 mm are retained to capture the contour of each rivet head, as shown in Figure 3b. After that, outliers are removed using the statistical filtering method, and the DBSCAN algorithm is applied to the filtered point cloud data. The initial DBSCAN clustering is performed using a neighborhood radius ε of 6 mm and a minimum point count MinPts of 500. The radius ε is selected to match the dimensions of the rivet head. A smaller radius tends to fragment the head into multiple small clusters and thus fails to capture it as a whole. Conversely, a larger radius may incorporate redundant points from adjacent rivet heads or regions far from the target head into the same cluster. The selected value for MinPts serves to suppress small-scale noisy clusters while avoiding over-filtering of the target rivet head region. If necessary, secondary DBSCAN clustering is then applied with a smaller ε of 4.5 mm and a larger MinPts of 800, which can further refine the segmentation and separate loosely connected rivet heads. In this way, the Rivet Head Clusters (RHCs) described by point clouds can be extracted as shown in Figure 3c. Finally, the Axis-Aligned Bounding Boxes (AABBs) are figured out for all clusters and expanded to the same dimension, as shown in Figure 3d. Each rectangular bounding box contains the DIRH and can be mapped onto a cylinder according to its cross-sectional size and height. Through these means, the DIRH can be extracted from the sampling points of the plate within the outlines of each cylinder. Figure 3e shows the extracted DIRH that may contain plate information surrounding the rivets.

### 2.3. Data-Reduction Algorithm

The DIRH extracted from the plate data typically contain no fewer than 8000 sampling points. To improve detection efficiency, it is advisable to reduce the number of points in the rivet head dataset reasonably. Therefore, the NMECM based on eigenvalues is adopted to simplify the DIRH. This method requires calculating the curvature of each point *p_i_* (*x_i_*, *y_i_*, *z_i_*) in the DIRH. Specifically, the *m* nearest neighbors of a given point are queried using a K-D tree, forming a discrete point set ***P***. Secondly, a covariance matrix is constructed based on ***P***. The matrix eigenvalues may reflect the second-order rate of change of the contours of the riveted head near the point. Then, the NMECM is formulated using Equation (1) so that the local surface characteristics can be fully captured, where *C_pi_* represents the curvature at point *p_i_*, and *λ*_1_, *λ*_2_, and *λ*_3_ are the matrix eigenvalues arranged in ascending order. The meaning of a non-maximum eigenvalue is that in the calculation of the curvature *C_pi_*, the numerator of Equation (1) is defined as the sum of *λ*_1_ and *λ*_2_, rather than the maximum eigenvalue *λ*_3_ alone. Finally, the curvature values are sorted in descending order, and the first 3000 points are selected to generate the Reduced Rivet Head Dataset (RRHD).

(1) $$C_{p}=\frac{\lambda_{1}+\lambda_{2}}{\lambda_{1}+\lambda_{2}+\lambda_{3}}$$

**Figure 4:**
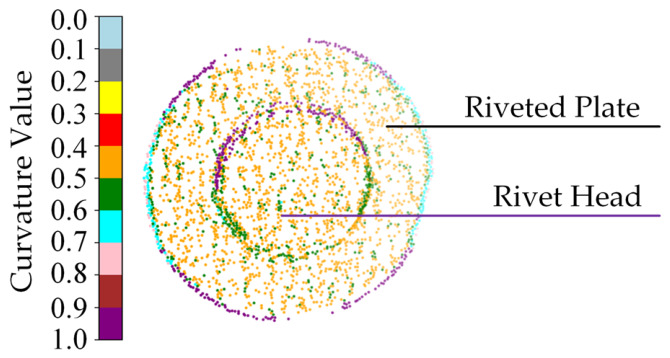
Color mapping of curvature values on the rivet head and surrounding plate surface point cloud. Different colors represent different curvature magnitudes, providing a visual reference for point cloud simplification.

Table 2 presents the RRHD generated using different curvature calculation methods. The three presented rows correspond to the defects of head damage, rivet rollover, and head protrusion based on the RRHD, respectively. The relationship between curvature values and colors is shown in Figure 4. As for Gaussian curvature, principal curvature, and mean curvature, it is evident that the generated RRHDs exhibit an incomplete description of the head defects and plate information surrounding the heads. The reason is that the principal curvature method is susceptible to noise interference, and the remaining two methods cannot effectively balance both the global and local information. By introducing the first two maximum eigenvalues, more abundant defect features can be retained. Therefore, the NMECM can facilitate feature learning and extraction for the subsequent recognition network. In addition, the selection of 3000 points is a comprehensive trade-off among three considerations: rivet head diameter, computational efficiency, and key geometric feature extraction for various head defects with different severity levels. The results of the reduction algorithm confirm that this choice is sufficient to preserve the key features of rivet heads. Decreasing the point count further leads to the noticeable loss of fine-grained details, particularly for mild defects. Increasing the count introduces mostly non-critical feature points, which yields no substantial improvement in accuracy but significantly increases computational overhead.

## 3. Methods

This section introduces the proposed PointGhost framework and describes its key modules. A flushness analysis method is also presented to quantify protrusion and indentation defects.

**Figure 5:**
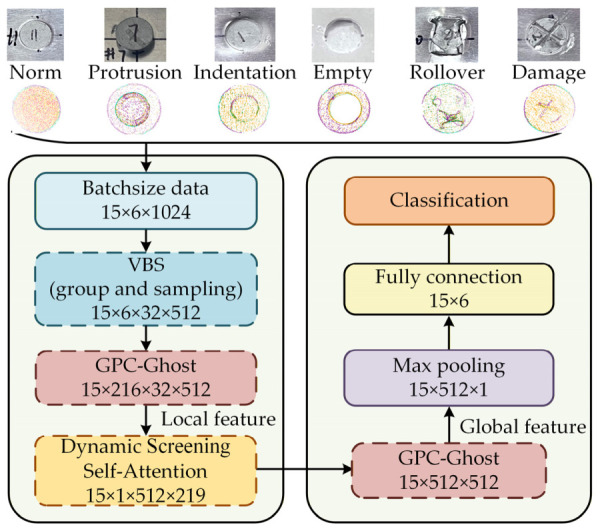
The PointGhost network architecture diagram. The numbers shown in each module indicate the corresponding dimensions.

This study proposes an efficient and lightweight point-cloud-classification network named PointGhost, built upon PointNet++. Its architecture is shown in Figure 5. The network consists of a lightweight sampling module and a feature-extraction module to perform defect classification. Based on the classification results, the flush measurement is carried out for rivets with protrusion or indentation defects. The distance is calculated between the highest point of the rivet head and the upper plate surface.

### 3.1. The PointGhost Network

The RRHD is processed in batches, with each batch containing 15 samples of the rivet head. Based on the farthest point sampling, 15 × 1024 data points are obtained to facilitate a subsequent performance comparison and analysis. Subsequently, the lightweight VBS mechanism is employed for efficient data grouping and sampling.

In the feature learning module, a GPC-Ghost is first introduced to efficiently learn local features and reduce computational costs. To address the issues of feature homogenization and insufficient information interaction caused by grouped pointwise convolution, a DSSA mechanism is proposed to enhance the model’s capability of geometric feature extraction. Subsequently, the extracted features are fed back into the GPC-Ghost module again to extract deeper features. In this manner, global feature learning can be achieved for multi-type defects of the rivet head, thereby providing richer feature information for subsequent classification and recognition.

Finally, max pooling is adopted to aggregate and extract global features for defect classification of the rivet head. The overall architecture adopts a hierarchical design concept, in which local and global feature extraction are performed in a single alternating manner. This approach avoids the redundant sampling operations of traditional methods, thereby improving both computational efficiency and feature extraction capability.

#### 3.1.1. Sampling Module

The VBS mechanism is adopted to construct the sampling module of the PointGhost network. This method divides the RRHD into blocks and generates regular virtual grid points. These virtual points are then mapped to their nearest real points. In this way, the task of uniform and efficient global point cloud sampling can be accomplished.

In the first step of VBS, spatial partitioning is performed with a cuboid employed as the basic partitioning unit. During this process, the spatial extent of 3D point cloud data, defined as [*x*_min_, *x*_max_] × [*y*_min_, *y*_max_] × [*z*_min_, *z*_max_], is partitioned into regular cuboid sub-blocks according to the prescribed block numbers *B*_x_, *B*_y_, and *B*_z_ along each dimension. The 3D dimensions of each sub-block are defined as follows:

(2) $$\Delta x=\frac{x_{\max}-x_{\min}}{B_{x}},\;\Delta y=\frac{y_{\max}-y_{\min}}{B_{y}},\;\Delta z=\frac{z_{\max}-z_{\min}}{B_{z}}$$

The index (*I*, *J*, *K*) of the sub-block to which any point *p_i_* belongs is presented as follows:

(3) $$I=\left\lfloor \frac{x_{i}-x_{\min}}{\Delta x}\right\rfloor ,\;J=\left\lfloor \frac{y_{i}-y_{\min}}{\Delta y}\right\rfloor ,\;K=\left\lfloor \frac{z_{i}-z_{\min}}{\Delta z}\right\rfloor$$

where min and max represent the minimum and maximum values, respectively. *I*, *J*, and *K* denote the axial offset indices of the sub-block to which point *p_i_* belongs, and ⋅ denotes the ceiling function.

The second step of VBS is to generate a regularly distributed virtual point set ***V****_IJK_* for each sub-region. The virtual points within this set are generated based on the geometric center of the current sub-block and can be determined according to the sub-block index and the number of points distributed within the sub-block. The coordinates of an arbitrary virtual point *v_IJK_* are given by the following:

(4) $$v_{IJK}=(x_{tc}+I\cdot \frac{\Delta x}{N_{tx}},y_{tc}+J\cdot \frac{\Delta y}{N_{ty}},z_{tc}+K\cdot \frac{\Delta z}{N_{tz}})$$

where (*x_tc_*, *y_tc_*, *z_tc_*) represents the geometric center coordinates of the *t*-th sub-block, and *N_tx_*, *N_ty_*, and *N_tz_* denote the respective number of point clouds distributed along the three coordinate axes within the sub-block.

The last step is to find the nearest neighbor data point *p*_min_. The distances between each virtual point and every real data point *p* within the same sub-block is calculated. The closest data point from the real point set *P_IJK_* is selected for single-point sampling and *p*_min_ can be expressed as follows:

(5) $$p_{\min}=\underset{p\in P_{IJK}}{\mathrm{argmin}}{\left\|p-v_{IJK}\right\|}_{2}$$

where *p* is a point in the point set *P_IJK_*

The entire sampling set *S* can be derived as follows:

(6) $$S=\overset{B_{x}}{\underset{I=1}{\cup }}\overset{B_{y}}{\underset{J=1}{\cup }}\overset{B_{z}}{\underset{K=1}{\cup }}\left\{\underset{p\in P_{IJK}}{\arg\;\min}{\left\|p-v_{IJK}\right\|}_{2}\left|v_{IJK}\in V_{IJK}\right.\right\}$$

The proposed sampling algorithm is compared with the curvature-based sampling algorithm, the random sampling algorithm, and the farthest point sampling algorithm based on the same test platform. The corresponding sampling times for each method are shown in Table 3. Among them, the VBS method achieves an average runtime of 0.005 s, which is 37 times faster than the 0.187 s required by the farthest point sampling algorithm.

#### 3.1.2. Feature-Extraction Module

**Figure 6:**
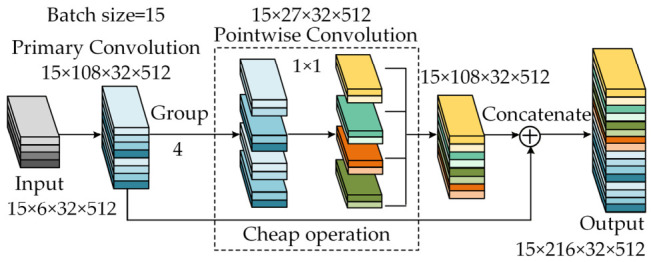
The GPC-Ghost model architecture for local feature extraction. The module consists of primary convolution, cheap operations, and feature concatenation, with the input and output feature dimensions indicated in the figure. The symbol “⊕” denotes the concatenation operation.

In the feature-extraction module, a lightweight model GPC-Ghost is first employed to perform efficient local feature learning. Its internal structure and dimensions are shown in Figure 6, where the block numbers *B*_x_, *B*_y_, and *B*_z_ are all set to 8 with 32 data points selected from each group. This module introduces Grouped Pointwise Convolution (GPC) to enhance the inexpensive operation part of the Ghost module, replacing traditional depthwise separable convolution to improve computational efficiency. In GPC-Ghost, the first part is the primary convolution operation. It uses standard convolution to generate primary features, consistent with the function of Ghost module. Subsequently, GPC is applied to these primary features to enhance the feature information. By adjusting the grouping strategy, a balance can be achieved between the computational cost and the feature representation capability. Finally, the primary features and the features processed by the inexpensive operation are concatenated to produce the final output. Compared with the original Ghost module, GPC allows flexible control over the number of groups to accommodate different computational requirements. It reduces the computational cost while maintaining feature-extraction capability. This model effectively improves feature learning performance and avoids excessive parameters and computational cost in traditional convolution modules.

**Figure 7:**
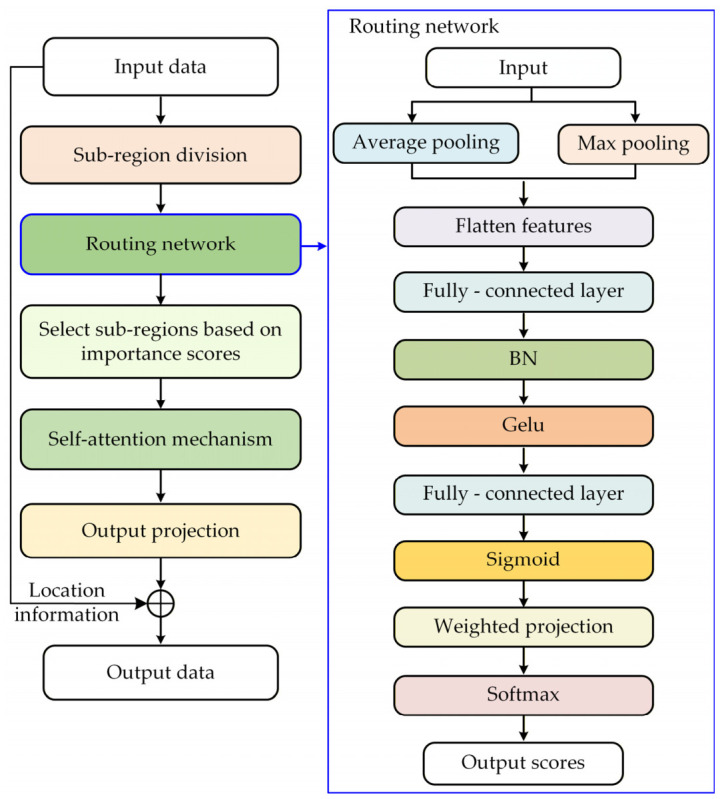
Diagram of DSSA mechanism architecture. The symbol “⊕” denotes the concatenation operation.

The second step in the module is to introduce a self-attention mechanism to further enhance feature representation capability. By establishing long-range dependencies between different features, it addresses the issues of feature homogenization and insufficient information interaction caused by the GPC operation in the GPC-Ghost model. To this end, an efficient DSSA mechanism is proposed, with its architecture illustrated in Figure 7. This approach can overcome the drawbacks of traditional self-attention mechanisms, namely the high computational complexity and large memory consumption when processing long-sequence information.

The DSSA module is implemented based on the constructed routing network. This network is derived from routing strategies that adaptively select and allocate important information. In this way, it can improve the model’s efficiency and performance in processing complex feature data. In the application of this mechanism, the output of GPC-Ghost model is divided into multiple sub-regions, and the routing network is used to compute an importance score for each sub-region. First, the routing network simultaneously performs global average pooling and global max pooling on each sub-region to extract statistical characteristics of the feature distribution. The results of the two pooling operations are concatenated along the channel dimension, and a two-layer fully connected network is applied to reduce dimensionality and extract key features. Specifically, the first fully connected layer extracts representative features via dimensionality reduction, while the second further reduces dimensionality to focus on key features. To facilitate more effective learning and the discrimination of complex patterns and relationships within the input data, the GELU activation function is inserted between the two layers to enhance the model’s nonlinear representation capability. Through this process, progressive feature optimization can be achieved and more compact feature information can be obtained for subsequent classification. Finally, the compressed features are projected using a weighted projection matrix, with the output dimension matching that of the sub-regions. The Softmax function (7) is then applied to generate the importance score *S*, which indicates the important feature paths to be selected.

(7) $$S=\mathrm{Softmax}(W_{2}\cdot \mathrm{Dropout}(\mathrm{GELU}(F_{p,s}{\left(W_{1}\right)}^{T})))$$

where *W*_1_ and *W*_2_ are learnable parameters, and *F_p_*_,*s*_ denotes the sub-region characteristic.

Based on the output importance scores *S* arranged in descending order, the routing network selects the top *k* sub-regions (typically *k* = 4), which serve as the input for the subsequent self-attention mechanism. Within this mechanism, the attention scores are computed based on the selected sub-regions. This computation is limited to interactions among the sub-regions, rather than performing global correlation calculations over the entire input sequence. Finally, output projection is performed on the attention scores, and the result is concatenated with spatial position information to enrich its geometric features. The final output is then fed into the second GPC-Ghost module for global feature learning.

### 3.2. Quantification of Rivet Head Flushness

**Figure 8:**
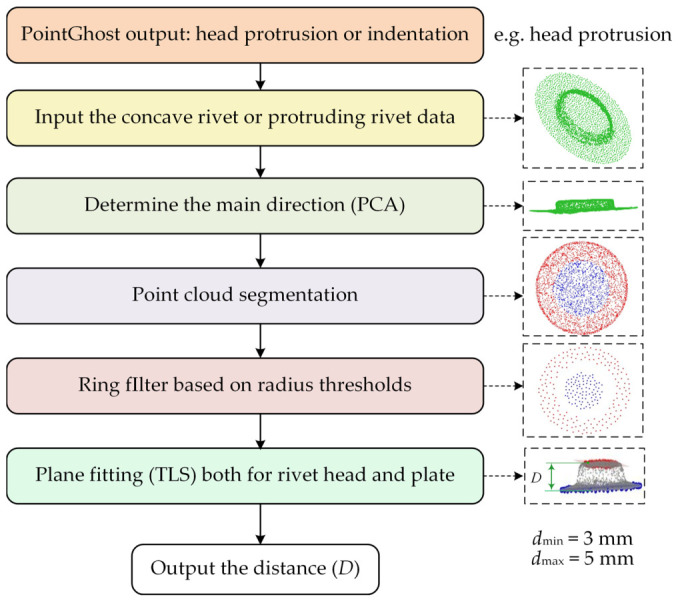
Quantification process of rivet flushness. The procedure includes PCA-based main direction determination, point cloud segmentation, ring filtering based on radius thresholds, TLS plane fitting for the rivet head and plate surfaces, and a final flushness calculation.

After classifying the multi-type rivet head defects based on the PointGhost network, it is necessary to quantify the rivet flushness for the defects of protrusion and indentation, respectively. The quantified flushness refers to the maximum distance from the rivet head surface to the surface of the riveted plate. For a qualified rivet joint, the quantified flushness value does not exceed ±0.3 mm. Based on the quantified height value, the two types of defects are further subdivided into three levels of severity: mild, moderate, and severe. This quantification process is based on the PCA method and plane fitting algorithm based on the TLS, as shown in Figure 8.

For the identified protruding and indented rivet samples, PCA is used to determine the principal direction of the rivet head. Since the curvature information has already been computed, this step incurs no additional computational cost. Second, the DIRH is divided into two parts along its perpendicular direction: one part characterizes the surface contour of the rivet head, and the other part characterizes the surface contour of the upper plate. During the riveting process, local deformation frequently occurs at the edges of the rivet head due to mechanical stress. This deformation may affect the plane fitting of both the rivet head and the plate during flushness quantification. For this reason, the RRHD is used to calculate the center point of the rivet head. An annular filtering region is then defined with respect to the center. The minimum filtering radius *d*_min_ and maximum filtering radius *d*_max_ are both set to eliminate edge data points within this region. Finally, the TLS method is applied to fit the respective plane of the rivet head and plate. The filtered rivet head data are then projected to the fitted plane of the rivet head. The flushness quantification value is calculated as the maximum distance from the fitted plane of the plate to the projected rivet head data. To verify the plane fitting performance, Table 4 presents the Mean Square Error (MSE) of the distances from the two sets of points to their respective fitted planes. The maximum MSE is 1.39 × 10^−3^, which meets engineering inspection requirements. Moreover, comparisons with RANSAC and the Least Squares (LS) method validate the effectiveness and efficiency of the proposed method. This quantified method is effective for assessing the severity of rivet defects.

## 4. Experiments and Results

This section presents the experimental evaluation of the proposed method. It includes the dataset description, classification performance analysis with an ablation study, rivet head flushness quantification, and detection performance analysis.

### 4.1. Rivet Head Dataset and Experimental Environment

**Figure 9:**
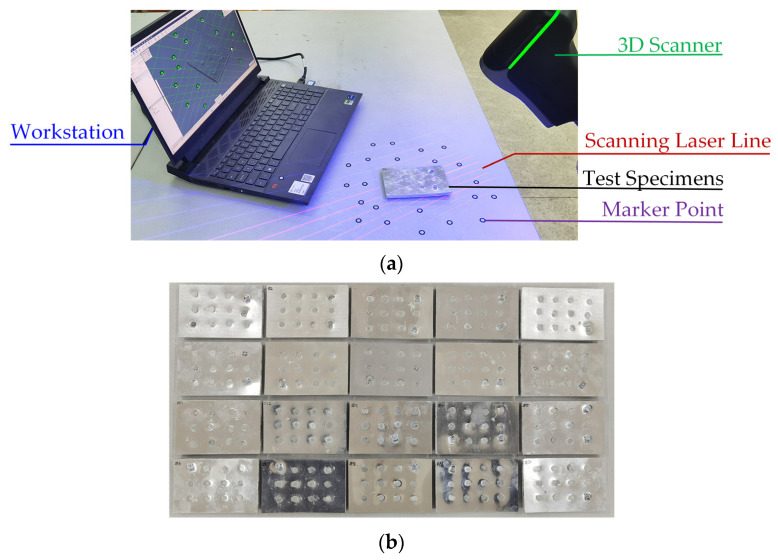
Experimental platform and specimens used for point cloud acquisition. (**a**) Experimental platform including the 3D scanner, workstation, marker points, and scanning laser lines; (**b**) riveted plate specimens containing different defect types.

All riveted specimens are scanned by a KSCAN-Magic combined handheld 3D scanner with a scanning accuracy of 0.05 mm, as shown in Figure 9a. Figure 9b shows a partial riveted specimen. Using the aforementioned data preprocessing algorithm, individual rivet head data are extracted with the number of subregions *n* set to 20. This value is chosen according to the specific dimensions of the specimens, as well as the size and number of rivets, to ensure the completeness of the rivet head data extracted after plane fitting. The data reduction is performed with the number of nearest neighbors *m* set to 30, which can effectively characterize the local geometric features between each given point and its neighborhood. The constructed dataset includes six types of rivet head states listed in Table 5. Among them, rivet head protrusions and indentations are further classified into three severity levels. A total of 1680 sample data sets is collected, of which 1260 are used for training, while the remaining 420 are reserved for testing.

The experimental environment is configured with an Intel Xeon Silver 4210 CPU and an NVIDIA RTX A5000 GPU. The software environment comprises Windows 10, Python 3.9, and PyTorch 2.1.0, with computations accelerated by CUDA 12.2 and cuDNN 8.8.1. The batch size for training is configured as 15, with six classified categories. The model is trained for 100 epochs. Each rivet data sample consists of spatial coordinates and normal vectors, with 1024 input points. Training is conducted with an initial learning rate of 0.001 using the Adam optimizer. The CPU utilization rate of the proposed method is approximately 5%, and the memory consumption reaches 2.3 GB at runtime.

### 4.2. Comparison of Defect Classification Performance

To examine the performance of the proposed network, a comparative performance analysis of different types of lightweight models, attention mechanisms, and point cloud classification networks is conducted under the same experimental environment, dataset, and parameter settings. Given the imbalanced class distribution, the Mean Accuracy (MA) metric is adopted to objectively reflect the overall balance of the network model in classification. Unlike the Overall Accuracy (OA), the MA is more conservative and provides a more effective indication of the model’s ability to recognize minority classes.

#### 4.2.1. Performance Comparison of Lightweight Models

**Figure 10:**
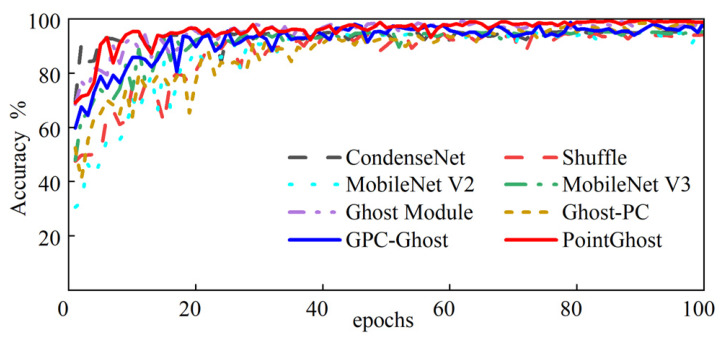
Training process and accuracy of eight lightweight modules integrated into PointNet++.

The training performance of the GPC-Ghost model and the resulting PointGhost network is compared with that of several lightweight models, including CondenseNet, ShuffleNet, MobileNet V2, MobileNet V3, Ghost, and Ghost-PC. For a fair and consistent comparison, each model is integrated into PointNet++ by replacing its original feature learning module. Table 6 presents the comparison results of training performance, and Figure 10 shows the training process along with the mean classification accuracy over epochs.

Compared with the PointNet++, CondenseNet shows no significant performance improvement. ShuffleNet reduces the computational cost and parameter count by 0.33 G and 0.23 M, respectively, but does not improve the MA. MobileNet V2 and MobileNet V3, owing to the introduction of expansion layers, incur a significant increase in both computational cost and parameter count without substantial improvements in accuracy. The Ghost module reduces the computational cost and parameter count by 0.43 G and 0.42 M, respectively, while increasing the mean accuracy by 3.8%. It demonstrates its lightweight effectiveness. Ghost-PC also reduces the computational cost and parameter count along with improved classification accuracy. However, its training process is unstable. The GPC-Ghost module reduces the computational cost and parameter count by 0.66 G and 0.59 M, respectively, achieving a 4.31% improvement in MA. It indicates a substantial performance gain. Among all compared models, the PointGhost network achieves the largest reductions in the computational cost and parameter count, namely 0.75 G and 1.22 M, respectively. It attains a mean classification accuracy of 99.86% and exhibits the most stable training behavior. The above comparison demonstrates that the lightweight design of GPC-Ghost module can effectively elevate the classification accuracy of the PointGhost network.

#### 4.2.2. Performance Comparison of Attention Mechanisms

The performance of five attention mechanisms—EMA, CBAM, CPCA, CA, and SA—is compared with that of DSSA. To ensure consistency, all six attention mechanisms are individually integrated into the feature learning module of PointNet++. Their respective impacts on defect classification performance are then comparatively analyzed.

**Figure 11:**
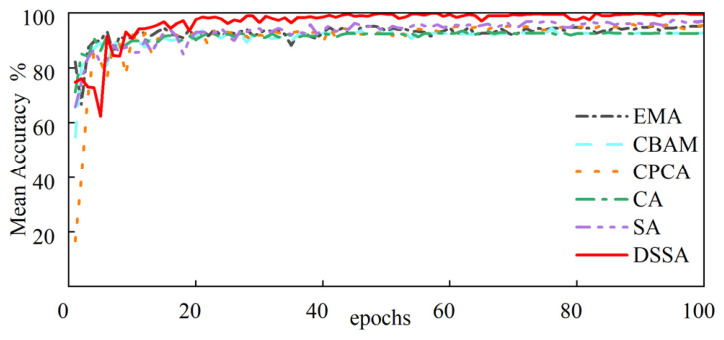
Training process and accuracy of six attention mechanisms integrated into PointNet++.

Table 7 presents the training comparison results for each attention module. The corresponding MA curves are presented in Figure 11. In the comparison with the results of PointNet++ summarized in Table 6, the introduction of attention mechanisms leads to consistent improvements in the model’s MA. Among them, the EMA attention mechanism increases the computational load due to the introduction of an additional fully connected layer, yet classification accuracy shows no significant improvement. The CBAM attention mechanism combines channel and spatial attention, which increases convolution operations. However, the increased operations fail to enhance global feature learning capability, leading to reduced classification accuracy and increased computational cost. The CA attention mechanism primarily analyzes and processes information across two spatial dimensions, resulting in a 1.29% decrease in classification accuracy.

In addition, compared with the results of PointNet++ in Table 6, the CPCA and SA modules improve MA by 0.98% and 2.6%, respectively. However, both modules significantly increase the computational cost and parameter count, thereby increasing the network’s computational burden. Although the self-attention mechanism of the SA module effectively captures global dependencies and enhances feature representation, this global learning strategy can result in high computational complexity when processing large input data. The comprehensive comparison reveals that the DSSA module achieves the highest mean classification accuracy of 99.44%. It shows the most significant performance improvement without a noticeable increase in the computational cost or parameter count. Furthermore, DSSA exhibits faster convergence during training and attains optimal classification accuracy in fewer epochs. In summary, the proposed DSSA attention mechanism effectively enhances feature extraction and classification performance through its dynamic screening self-attention mechanism. In addition, its training process is stable with fast convergence, making it more suitable for lightweight network design.

#### 4.2.3. Performance Comparison of Classification Networks

**Figure 12:**
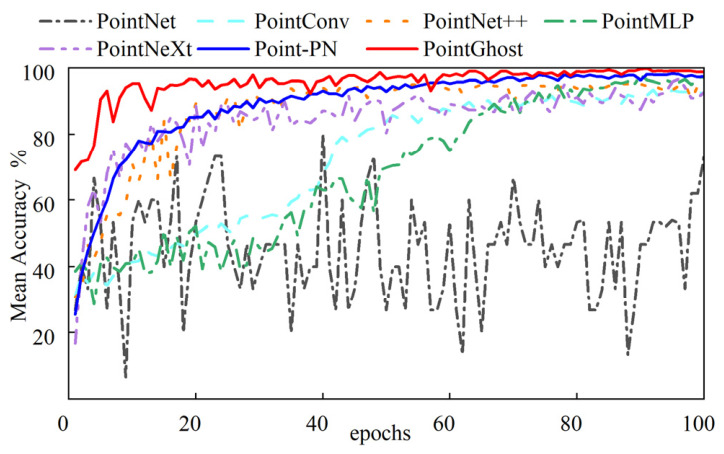
Training process and accuracy of seven classification models.

To benchmark the performance of different types of point cloud classification networks, the PointGhost network is trained alongside PointNet, PointConv, PointMLP, PointNet++, PointNeXt, and Point-PN on the constructed dataset. Each network is trained for ten rounds, with 100 epochs per round. To optimize training performance, the batch size for PointMLP and PointNeXt is set to 20, and the learning rate for PointMLP is set to 0.1. Table 8 presents the MA of each classification network across all training rounds. The performance gap represents the difference in MA between each classification network and PointGhost. The downward arrow (↓) denotes inferior performance relative to PointGhost. Figure 12 shows the training process corresponding to the best accuracy achieved by each network.

An analysis of the training results indicates that PointNet exhibits an unstable training process with significant fluctuations, resulting in the lowest classification accuracy among all evaluated networks. In contrast, PointConv and PointMLP achieve slightly improved mean classification accuracy but require longer training times. PointNet++, PointNeXt, and Point-PN exhibit relatively smaller fluctuations in classification accuracy across rounds, indicating better stability. However, they still have limitations in learning and extracting subtle defect features, making it difficult to meet the requirements for different defect detection of rivet heads.

The comprehensive comparison shows that the PointGhost network achieves superior training performance, with an MA of 99.49%. This surpasses Point-PN and PointNeXt by 0.93% and 4.55%, respectively. Moreover, it maintains stable classification accuracy, with the majority of test results exceeding 99%. It can be seen from its stable training process and consistent classification performance that the network exhibits good robustness. Compared with PointNet++, the MA is improved by 4.41%, which preliminarily verifies the effectiveness and reliability of the proposed network for point cloud data classification task.

An ablation study is conducted to further evaluate the effectiveness of each key component in PointGhost. As shown in Table 9, the introduction of VBS significantly reduces the inference time from 107.82 ms to 32.68 ms, while maintaining comparable classification performance. This indicates that it is effective in accelerating point cloud sampling. After integrating GPC-Ghost, the parameter count is reduced from 1.467 M to 0.211 M, while the classification accuracy is significantly improved. This shows that the lightweight feature extraction design is efficient. With the addition of DSSA, the final PointGhost framework achieves the best classification performance, with an F1-score of 99.50% and a MA of 99.72%. This confirms the effectiveness of the proposed attention mechanism.

**Figure 13:**
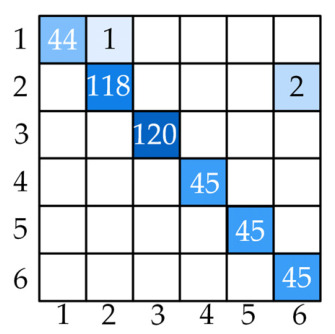
Confusion matrix of the proposed PointGhost network.

Figure 13 presents the confusion matrix of PointGhost on the test set, providing an intuitive visualization of its classification performance. In the figure, the normal rivet head, head protrusion, head indentation, rivet rollover, empty rivet, and head damage are labeled as 1 through 6, respectively. These results indicate that PointGhost achieves strong classification performance across different rivet defect categories. Only three misclassifications are observed, further demonstrating the effectiveness and stability of the proposed method.

**Figure 14:**
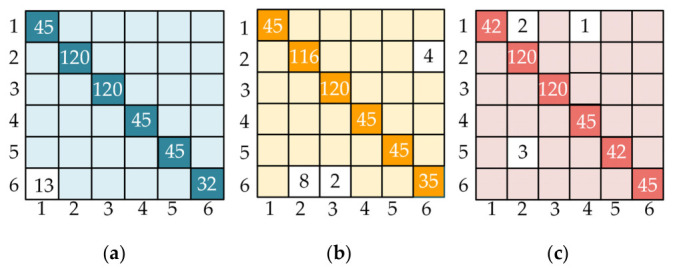

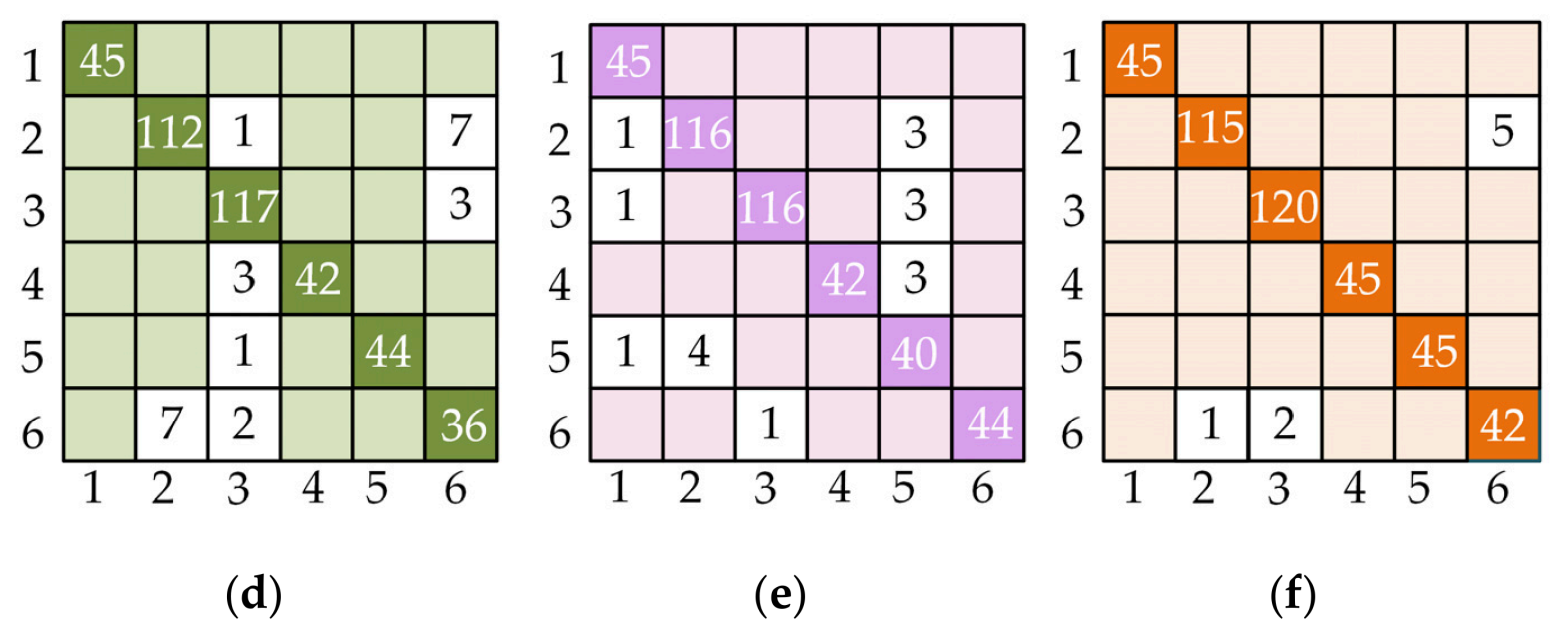
Misclassification results of six point cloud classification networks. (**a**–**e**) Show the minimum misclassification cases of PointNet++, PointConv, PointMLP, PointNeXt, and Point-PN, respectively, while (**f**) shows the maximum misclassification case of PointGhost.

To further evaluate the classification performance and generalization capability of the PointGhost network, tests are conducted based on the test set (420 data groups) using the classification networks trained in Table 8. The results are presented in Table 10 and Figure 14. For PointGhost, the misclassification results corresponding to the maximum misclassification rate are shown, whereas for the other five network models, the results with the lowest number of misclassifications are displayed. In the figure, the normal rivet head, head protrusion, head indentation, rivet rollover, empty rivet, and head damage are labeled as 1 through 6, respectively.

The comparison shows that PointNet++ achieves an average misclassification rate of 3.29%. The result corresponding to its minimum misclassification rate shows that all 13 head damage samples are misclassified as normal heads. This failure is due to the network’s inadequate learning of both the normal vector features of point cloud data and the characteristics of head damage. Although the model achieves a low overall misclassification rate, such misclassifications—labeling defective rivets as normal—would severely degrade riveting quality inspection performance. PointNeXt yields the highest average misclassification rate of 7.83%. The misclassification patterns are diverse, including head protrusions, indentations, empty, head damage, and rollover. For instance, among eight head protrusion samples, one is misclassified as head indentation, and the remaining seven are misclassified as head damage. This indicates that PointNeXt fails to effectively extract and learn the subtle features of different defect types, resulting in poor discrimination between samples with similar local geometric features but different defect categories. Point-PN, PointConv, and PointMLP show progressively lower misclassification rates relative to PointNeXt, indicating improved feature-learning capability. In contrast, PointGhost achieves the lowest average misclassification rate of 1.19%, with a maximum rate of 1.9%. Although this maximum slightly exceeds PointMLP’s minimum misclassification rate of 1.43%, PointGhost maintains a lower overall misclassification level. An analysis of the test result with the minimum misclassification rate reveals that only one head protrusion sample is misclassified as head damage. An analysis of the test result with the maximum misclassification rate shows that, among the eight misclassified samples, five head protrusions are misclassified as head damage, one head damage is misclassified as head protrusion, and two head damage are misclassified as head indentation. These misclassifications arise because the fine geometric features of head defects tend to hinder the model from adequately learning their distinct morphological characteristics. This limitation can be addressed by incorporating additional training samples that exhibit such subtle defect features. The above analysis demonstrates that the PointGhost model achieves superior overall test performance. This is attributed to its stable training process and consistent classification behavior, which further indicate the network’s strong robustness and generalization ability. These analytical results validate that PointGhost can perform the classification of multi-type rivet head defects efficiently and accurately.

### 4.3. Quantification of Rivet Defect Severity

Following rivet head state classification, flushness quantification is performed on rivet head samples with protrusion and indentation defects. It can be achieved by calculating the maximum distance from the fitted plane of the riveted plate to the projected filtered rivet head data on the fitted plane of the rivet head.

Table 11 presents the quantified flushness results. Among them, two misclassifications are identified: a mild protrusion defect with a quantified value of 0.62 mm and a mild indentation defect with a quantified value of 0.61 mm, both of which are incorrectly classified as moderate defects. The quantified values deviate from the upper bound of the mild defect range by 0.02 mm and 0.01 mm, respectively. Overall, the quantified results of the proposed method are highly consistent with actual measured values. Among the 240 samples, only two cases are misjudged, yielding a misclassification rate of 0.83%. No significant deviations were observed, thus verifying the feasibility and effectiveness of the proposed quantification method for efficient defect severity assessment.

Finally, the inspection process for an individual rivet comprises four sequential stages: individual rivet data extraction, rivet head point cloud data reduction, head status classification, and head flushness quantification (only for protrusion and indentation). The average processing times for these stages are 0.61 s, 0.64 s, 0.02 s, and 0.03 s, respectively. Excluding the sampling time, the total time from data processing to classification and quantification for a single rivet is within 1.5 s. This demonstrates efficient processing and reliable classification performance, making it suitable for industrial inspection applications.

### 4.4. Analysis of Detection Performance

The detection performance of the proposed method is governed by three factors: limitation of scanning accuracy, impact of data reduction, and influence of residual noise. Firstly, all riveted specimens are scanned using a 3D scanner with a nominal scanning accuracy of 0.05 mm. However, the actual detection accuracy of the system is expected to be no better than 0.15 mm under extreme conditions (e.g., unfavorable scanning angles or elevated noise levels), i.e., at least 3 times lower than the nominal sensor accuracy. Secondly, although the RRHD preserves the primary defect features of the riveted heads and significantly enhances detection efficiency, it inevitably removes some fine-scale geometric information, thereby limiting the detection capability for small defects. Finally, under extreme scanning conditions, some noise points may persist despite the application of filtering algorithms. These residual noise points may cause misclassifications, especially for very small defects where the signal-to-noise ratio is low. As for industrial application, parallel computing techniques can be utilized to achieve the concurrent detection of each rivet head. In this way, the presented method is suitable for scenarios where the total inspection time, including the scanning time, is no less than 40 s, such as a quality inspection island in automotive production lines.

## 5. Conclusions

This research aims to address the challenges of complex defect feature extraction from rivet heads on an automotive body and the limited performance of conventional classification networks. The inspection method is investigated for five common types of rivet head defects. Riveted specimens are prepared for defect detection based on the point clouds of riveted plates collected using a 3D scanner. An individual rivet head dataset is created via extraction and reduction from the acquired point data of the plates based on the DBSCAN clustering method and the NMECM. To achieve efficient classification of multi-type rivet head defects, we propose PointGhost, a lightweight classification model that integrates a VBS mechanism, a GPC-Ghost network, and a DSSA mechanism. The model performs both local and global feature learning on the key geometric information of head defects, thereby enhancing defect feature representation and enabling accurate and efficient classification. Subsequently, the severity of rivet protrusion and indentation defects is quantified using PCA combined with the TLS plane fitting algorithm. On the custom-built dataset, the PointGhost model is validated for stable training and consistent classification performance with reasonable robustness. The model achieves a mean accuracy of 99.49%. Compared with PointNet, PointConv, PointNet++, PointMLP, PointNeXt, and Point-PN, the presented network achieves improvements of 24.32%, 2.47%, 4.41%, 3.68%, 4.55%, and 0.93%, respectively. A comparative analysis of test results reveals that PointGhost attains the lowest average misclassification rate of only 1.19%, outperforming PointNeXt, PointConv, Point-PN, and PointMLP by 6.64%, 3.83%, 3.41%, and 2.38%, respectively. Furthermore, the network requires only 0.13 G of computational cost and 0.25 M parameters and hence significantly improves computational efficiency. The total time for a single rivet from data processing to classification and quantification does not exceed 1.5 s, demonstrating efficient processing and reliable classification performance. This demonstrates its potential for industrial inspection applications. However, the proposed method shows limited effectiveness in identifying small defect features, primarily due to the difficulty of fabricating small-defect specimens, which results in an insufficient number of such samples in the dataset. Future research will focus on the fabrication and detection of mild defect samples with defect sizes ranging from 0.1 to 0.2 mm. By further refining and optimizing the network architecture and parameters, we aim to enhance detection performance for small defect samples, reduce the misclassification rate, and deploy the proposed detection method for industrial applications.

## Figures and Tables

**Table 1:** Types of rivet head.

Type Code	State Type	Defect Range (mm)	Severity Levels
1	normal	height difference: [−0.3, 0.3]	none
2	damage	minimum defect size: ≥0.3	none
3	protrusion	height difference: (0.3, 0.6)	mild
		height difference: [0.6, 0.9)	moderate
		height difference: ≥0.9	severe
4	indentation	height difference: (0.3, 0.6)	mild
		height difference: [0.6, 0.9)	moderate
		height difference: ≥0.9	severe
5	empty	-	none
6	rollover	-	none

**Table 2:** Different curvature calculation methods based on reduced rivet head dataset.

Original Data	Gaussian Curvature	Principal Curvature	Mean Curvature	Our Curvature
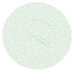	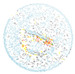	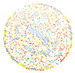	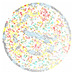	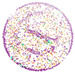
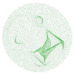	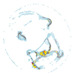	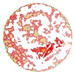	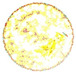	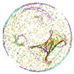
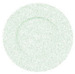	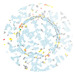	** 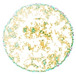 **	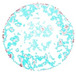	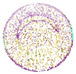

**Table 3:** Comparison of sampling algorithm times.

Sampling Algorithm	Normal	Rollover	Protrusion	Indentation	Damage	Empty	Average Time
Curvature-Based Sampling (s)	1.621	1.503	1.466	1.371	1.643	1.558	1.527
Random Sampling (s)	0.591	0.127	0.123	0.126	0.677	0.115	0.293
Farthest Point Sampling (s)	0.296	0.203	0.094	0.099	0.336	0.092	0.187
Ours (VBS) (s)	0.003	0.004	0.006	0.003	0.006	0.005	0.005

**Table 4:** Comparison of four algorithms for flushness quantification.

Plane Fitting Algorithm	Time (s)	Fitted Plane MSE-1	Fitted Plane MSE-2
RANSAC	16.86	5.09 × 10^−3^	6.47 × 10^−3^
PCA + RANSAC	1.49	3.61 × 10^−3^	3.70 × 10^−5^
PCA + LS	0.04	1.62 × 10^−3^	2.54 × 10^−5^
Ours (PCA + TLS)	0.02	1.39 × 10^−3^	3.45 × 10^−5^

**Table 5:** Rivet head dataset.

State Type	Defect Severity	Training Samples	Test Samples
normal	none	135	45
head protrusion	mild/moderate/severe	360	120
head indentation	mild/moderate/severe	360	120
rivet rollover	none	135	45
empty rivet	none	135	45
head damage	none	135	45
total samples	-	1260	420

**Table 6:** Performance comparison of lightweight models.

Method	MA (%)	FLOPs (G)	Paras (M)	Time (mins)
PointNet++	94.81	0.88	1.47	142
CondenseNet	95.19	0.77	1.46	133
ShuffleNet	95.05	0.55	1.24	131
MobileNet V2	95.19	3.09	3.21	339
MobileNet V3	95.19	3.11	4.12	346
Ghost	98.61	0.45	1.05	108
Ghost-PC	98.52	0.49	1.08	121
Ours (GPC-Ghost)	99.12	0.22	0.88	48
Ours (PointGhost)	99.86	0.13	0.25	43

**Table 7:** Performance comparison of attention mechanisms integrated into PointNet++.

Attention Mechanism	MA (%)	FLOPs (G)	Paras (M)
EMA	95.19	1.12	1.51
CBAM	94.44	1.11	1.49
CPCA	95.79	1.42	1.66
CA	93.52	1.45	1.68
SA	97.41	1.19	1.78
DSSA	99.44	0.91	1.52

**Table 8:** Mean accuracy comparison of classification models.

No.	PointNet	PointConv	PointNet++	PointMLP	PointNeXt	Point-PN	Ours
1	81.90	96.90	95.19	96.51	94.75	98.49	99.86
2	82.38	96.90	95.19	96.35	95.79	98.49	99.72
3	69.52	96.90	95.19	95.32	94.38	98.49	99.72
4	76.43	97.86	95.19	97.94	95.09	98.49	99.25
5	76.43	96.90	95.19	97.30	95.38	99.05	99.72
6	67.86	97.38	94.44	89.76	95.15	98.49	99.72
7	78.81	96.43	94.81	95.87	93.93	98.49	99.07
8	74.05	96.19	95.19	95.71	94.23	98.65	99.72
9	72.86	97.14	95.19	96.75	94.40	98.49	99.12
10	71.43	97.62	95.19	96.59	96.30	98.49	98.98
Mean	75.17	97.02	95.08	95.81	94.94	98.56	99.49
Rate	24.32↓	2.47↓	4.41↓	3.68↓	4.55↓	0.93↓	-

**Table 9:** Ablation study of PointGhost. √ denotes the use of the corresponding module, while × denotes its absence.

Model	VBS	GPC-Ghost	DSSA	MA (%)	Precision (%)	Recall (%)	F1-Score (%)	Params (M)	Time (ms)
PointNet++	×	×	×	95.19	96.26	95.19	95.08	1.467	107.822
+VBS	√	×	×	95.19	96.26	95.19	95.08	1.467	32.679
+VBS + GPC-Ghost	√	√	×	98.52	99.46	98.52	98.95	0.211	9.750
PointGhost	√	√	√	99.35	99.15	99.35	99.24	0.314	10.714

**Table 10:** Misclassification rate comparison of classification models.

Method	Min. Misclassifications	Max. Misclassifications	Min. Rate (%)	Max. Rate (%)	Avg. Rate (%)
PointNet++	13	17	3.10	4.05	3.29
PointConv	14	28	3.33	6.67	5.02
PointMLP	6	35	1.43	8.33	3.57
PointNeXt	24	41	5.71	9.76	7.83
Point-PN	17	25	4.05	5.92	4.60
PointGhost	1	8	0.24	1.90	1.19

**Table 11:** Quantitative results of the protrusion and indentation.

Defect Severity	Actual Count	Quantified Count	Range of Actual Distances (mm)
mild protrusion	40	39	0.34~0.59
moderate protrusion	40	41	0.61~0.88
severe protrusion	40	40	0.92~3.56
mild indentation	40	39	0.36~0.59
moderate indentation	40	41	0.61~0.87
severe indentation	40	40	0.91~2.03

## Data Availability

The data presented in this study are available on request from the corresponding author.
